# Pulmonary Edema Following Intrathecal Fluorescein Injection; a Case Report

**Published:** 2019-02-12

**Authors:** Faranak Behnaz, Masih Ebrahimy Dehkordy, Hamidreza Azizi Faresani, Mohammadreza Shahmohammadi

**Affiliations:** 1 Anesthesiology Department, Shohadaye Tajrish Hospital, Shahid Beheshti University of Medical Sciences, Tehran, Iran.; 2 Functional Neurosurgery Research Center, Shohadaye Tajrish Neurosurgical Comprehensive Center of Excellence, Shahid Beheshti University of Medical Sciences, Tehran, Iran

**Keywords:** Pulmonary edema, injections, spinal, fluorescein, emergency treatment, complications, cerebrospinal fluid leak

## Abstract

Intrathecal Fluorescein has been used widely for detection of cerebrospinal fluid (CSF) leakage. After administration of fluorescein many serious complications may happen. Pulmonary edema is one of the most serious complications that require emergency responses. In this study, we report a complicated case of pulmonary edema following Intrathecal fluorescein injection.

## Introduction

Intrathecal injection of a sodium fluorescein solution following a thorough endoscopic examination has been used to identify the site of cerebrospinal fluid (CSF) leakage (1, 2). Sodium fluorescein has a relatively low molecular weight and is a highly water-soluble compound. It is well tolerated by most patients, but its intrathecal injection is an invasive procedure with an associated risk of complications. Complication occurs in 5-10% of patients and ranges from mild to severe (3, 4). Severe reactions are not common, but laryngeal edema, pulmonary edema, anaphylaxis, status epilepticus, myocardial infarction and cardiac arrest have been reported as some of its complications (5-7). Non-allergic histamine release in the absence of antigen-antibody reaction (anaphylactoid reactions); vasovagal phenomenon resulting in bradycardia, arterial hypotension and reduced cardiovascular perfusion; immediate hypersensitivity reaction to the drug (anaphylactic reactions); anxiety-related medullary sympathetic discharge, eliciting tachycardia and myocardial stress; and direct vasospastic toxic effect of intravenous injection are among the proposed mechanisms for occurrence of complications (8-10). In this study, we report a complicated case of pulmonary edema following intrathecal fluorescein injection.

## Case presentation:

A 33-year-old man presented with 8-month history of intermittent cerebrospinal fluid (CSF) leakage from his nostril following removal of fringe body from his orbital cavity. In medical history, the patient had eye trauma, mild asthma, and was under treatment of glaucoma with Timolol eye drop. He was admitted to the operating room for trans-sphenoid endoscopic surgery. His preoperative blood pressure measured via noninvasive method was 135/80 mmHg and he had a pulse rate of 90/minute with normal respiratory rate and O2Saturation of 100% with oxygen.

Laboratory findings showed fasting blood sugar (FBS): 93 mg/dl, blood urea nitrogen (BUN): 20 mg/dl, creatinine: 0.8 mg/dl, sodium: 138 mEq/L, hemoglobin: 13.3 g/ dl, platelet: 260000 /microliter, and international normalized ratio (INR): 1. His imaging result was normal, and normal cardiovascular risk for operation was reported in pre-operation cardiology consultation.

He underwent cardiac and invasive blood pressure (IBP) monitoring, pulse oximetry, capnometry, and intake/output checking. Anesthesia was induced via Fentanyl (200 micg), Midazolam (2 mg), Lidocaine (80 mg), Propofol (200 mg), Cisatracuriom (18 mg), and then orotracheal intubation was done.

**Figure 1:**
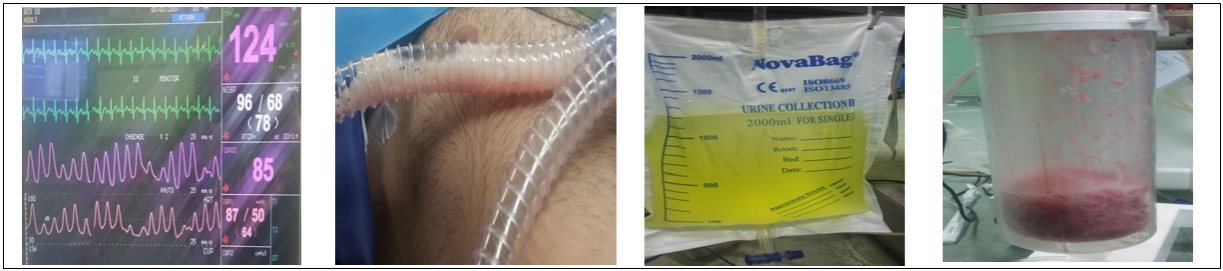
Changes observed 10 minutes after intrathecal fluorescein injection

After positioning of the patient, 0.5 cc of fluorescein 5% was mixed with 10 cc of the patient’s CSF and then re-injected via a lumbar puncture at the level of L4-L5 spinal column. After 10 minutes, the patient’s blood pressure dropped unexpectedly (IBP: 87/50 mmHg), and his pulse rate rose to 124/minutes and O2 saturation dropped to 85%. Shortly after this event, some pink foamy secretions appeared in the transparent circuiting tube of the anesthesia machine and suction bottle cavity and urine color changed to shiny green (figure 1).

Patient underwent immediate supportive vital managements including change of ventilator set up, medical administration of supportive drugs, and close vital signs and hemodynamic monitoring. 

The surgeon decided to postpone the surgery. The patient was directly transferred to intensive care unit (ICU) and ventilated with positive pressure mode. After one day, the intensivist decided to wean him from mechanical ventilation and he was extubated successfully. Finally, the patient was discharged from the hospital with good general condition after two days from his admission.

## Discussion:

The use of intrathecal fluorescein injection (in the subarachnoid space) for detecting the source of leakage, dates back to 1960, when Kirchner and Proud used this method to recognize and locate CSF fistulas in the cranial base (11). Fluorescein quickly diffuses out of the capillaries into the extravascular fluid compartments. 

In the circulation, fluorescein moves mostly bound to plasma proteins and is metabolized in the liver through glucuronidation. The monoglucuronide has about 4.5% of the fluorescence of free fluorescein, and both are excreted through the kidney. While most fluorescein is eliminated after 24 hours, it can still be traced in urine up to a week after its infusion (12).

 The usefulness of this test depends on the extent of the dural defect, rate of leakage, timing of the intrathecal injection, and rate of CSF turnover that could dilute or disperse the fluorescein. Reported complications of the solution’s injection, and thus limitations to its use, have ranged from mild to severe among which are tinnitus, headache, nausea and vomiting, transient pulmonary edema, confusion, seizures, and coma, and death. 

Guimaraes R et al. reported that when they used a low dose of fluorescein (0.25cc of 5% solution) and diluted it with CSF and injected the solution slowly, complications did not happen (13). 

The reasons for the complications were found in the method of administration, formulation of the solution, idiopathic reactions, and concentration or dose of fluorescein (13-15). 

Side effects after the administration of intravenous fluorescein are uncommon and mostly harmless. Reactions more commonly seen include nausea and occasional vomiting. Severe reaction following intravenous fluorescein injection was observed in a patient who had an anaphylactic reaction (8).

The exact mechanism of fluorescein-induced pulmonary edema following intrathecal injection is not known and it could happen due to multiple factors. Hypertension with overloading of the left ventricle and chemical alveolitis may cause pulmonary edema with fluorescein. A central neurogenic mechanism may play a role in the pulmonary changes (16).

Although our patient had no underlying heart disease, bronchopulmonary infection, or any other risk factor of pulmonary edema, intrathecal fluorescein injection could be considered as a causative factor for pulmonary edema. Anesthesiologists and medical practitioners should be aware of this serious adverse reaction of administrating this drug. 

## Conclusion:

Medical practitioners should be aware of the complications of intrathecal fluorescein administration.
